# Comparative Analysis of Fracture Resistance in Endodontically Treated Molars Using Lithium Disilicate and Composite Overlays Versus Endocrowns

**DOI:** 10.1155/ijod/5061799

**Published:** 2025-10-13

**Authors:** Sintija Miluna-Meldere, Baiba Springe, Evaggelia Papia

**Affiliations:** ^1^Department of Prosthetic Dentistry, Faculty of Dentistry, Riga Stradins University LV-1007, Riga, Latvia; ^2^Department of Materials Science and Technology, Faculty of Odontology, Malmö University 205 06, Malmö, Sweden

## Abstract

**Statement of Problem:**

Restoring extensively damaged, endodontically treated molars with an indirect restoration that both preserves and protects the remaining tooth structure is a significant challenge. Overlays and endocrowns offer a less invasive alternative compared to conventional crowns.

**Purpose:**

The aim of this study was to evaluate the fracture resistance and fracture patterns of molars restored with different materials and designs.

**Materials and Methods:**

This study involved 40 extracted human molars, which underwent endodontic root canal treatment and were divided into four groups: composite core build-up with pressed lithium disilicate overlay (OL) (*n* = 10); composite core build-up with milled composite overlay (OC) (*n* = 10); pressed lithium disilicate endocrown (EL) (*n* = 10); milled composite endocrown (EC) (*n* = 10). The teeth were subjected to thermocyclic loading (10,000 cycles between 5 and 55°C), followed by chewing simulation (0–50 N at 1.6 Hz for 600,000 cycles with a 10° inclination), another round of thermocyclic loading (10,000 cycles between 5 and 55°C), and finally, a fracture strength test (5 mm steel ball at a 10° inclination with a load rate of 0.5 mm/min). Afterwards methylene blue was used to stain any cracks or fracture lines in the teeth for microscopic evaluation.

**Results:**

The findings suggest that while the material type may not significantly impact fracture resistance or catastrophic fracture likelihood, the type of restoration (endocrown vs., overlay) is a crucial factor to consider.

**Conclusions:**

Clinicians should weigh the higher risk of catastrophic fractures associated with endocrowns when selecting restorative options for endodontically treated teeth.

**Clinical Implications:**

Overlays and endocrowns offer similar overall tooth fracture resistance, making both viable options for restoring endodontically treated molars. However, OLs may better withstand chewing forces and posed no risk of catastrophic tooth fractures, unlike ECs, which carry a higher tooth fracture risk.

## 1. Introduction

Endodontically treated teeth are considered more fragile than vital teeth due to changes in dentin structure and significant hard tissue loss [1–3], especially when marginal ridges are missing. To reinforce these weakened structures, restorations covering the tooth cusps are recommended, particularly when cusps are less than 3 mm thick [4–6].

Traditionally, crowns have been the gold standard for restoring such teeth [7, 8], offering superior survival rates compared to direct restorations [9, 10]. However, advancements in adhesive cementation have led to a shift toward more conservative options like overlays and endocrowns, both covering all tooth cusps [11, 12]. Despite various preparation techniques, no conclusive evidence favors one method over another [5, 13–18].

Clinicians often face challenges in selecting the most suitable restoration type. Composite core build-ups with overlays can improve stress distribution but are time-consuming [14]. Also, the use of reinforcement materials, such as glass fiber in the composite core, can enhance fracture resistance [19]. Endocrowns offer easier and quicker preparation but may increase internal stress due to their extension into pulp chamber and increased material thickness [16, 20, 21]. Additionally, preparation design, especially interproximal margin placement, significantly impacts fracture resistance and tooth longevity [22, 23].

After selecting the restoration type, choosing the material—lithium disilicate or composite resin is crucial. Lithium disilicate provides excellent optical and mechanical properties with high flexural strength, while composite resin is more cost-effective and easier to repair but has lower flexural strength [24–26]. Both materials demonstrate high survival rates over time [27, 28].

Considering the challenges from existing literature, the aim of this study was to evaluate the fracture resistance and patterns of endodontically treated molars restored with different material overlays and endocrowns. The null hypothesis was that there would be no difference in fracture resistance strength among four endodontically treated teeth groups restored with either composite overlay (OC), composite endocrown (EC), lithium disilicate overlay (OL), or lithium disilicate endocrown (EL). It was also hypothesized that all restorations, regardless of the material, would have the same fracture resistance.

## 2. Materials and Methods

### 2.1. Funding and Ethics Approval

This prospective cross-sectional in vitro study was funded by the Riga Stradiņš University “Student Research and Innovation Grant” and approved by its Ethics Commission on March 11, 2021 (Number 22-2/94/2021).

### 2.2. Inclusion of Samples

**Figure 1:**
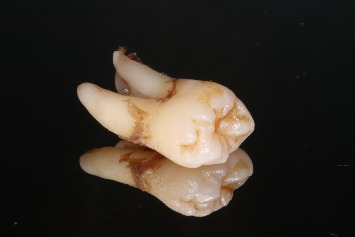
Extracted human molar according to inclusion criteria.

Fifty-five extracted molars, obtained from the Clinic of Oral and Maxillofacial Surgery, Riga Stradiņš University, with all patients providing consent for participation and material collection, were initially selected and stored in distilled water at room temperature. The inclusion criteria were as follows: extracted maxillary and mandibular molar teeth with remaining all intact roots, crown part with intact buccal and palatal/lingual walls including small occlusal/cuspal defects, and intact marginal ridges or shallow proximal defects (Figure 1). Each tooth was revised by removing any fillings or caries and then evaluated based on specific criteria: no visible cracks; intact buccal and palatal/lingual walls with permissible occlusal or cuspal reduction up to 1.5 mm; intact marginal ridges or proximal cavities at least 1.0 mm above the cementoenamel junction (CEJ); proximal cavity extension not exceeding 1.0 mm beyond the mesio or distobuccal, mesio or distopalatal/lingual line angles; the thickness of the functional cusps 2.5–3.0 mm and supporting cusps 2.0–3.0 mm (measured at the thinnest part); the depth of the pulp chamber minimum of 4 mm (measured from the proximal cavity to the pulpal floor). All measurements were performed using dental caliper (Iwanson, OR-7002) and periodontal probe. Teeth not meeting these criteria were excluded (*n* = 15).

### 2.3. Tooth Preparation

Forty (*n* = 40) extracted molars met the criteria for further research and were divided into two groups (mandibular and maxillary, *n* = 20 each). Endodontic root canal treatment was performed for each tooth with ProTaper Gold (Dentsply; Sirona) rotary files until number F2, respecting the root canal rinsing protocol: sodium hypochlorite solution 2.5% 2–5 mL (i-Dental), citric acid 10% 5 mL (i-Dental), distilled water 5 mL, and 2% chlorhexidine 5–10 mL. Subsequently the root canals were filled with guttapercha points (ProTaper Gold Conform fit; Dentsply Sirona) using dental sealant (Adseal; Biomed) and orifices were sealed with resin-based lining material (Ionofast; Biodinamica).

**Figure 2:**
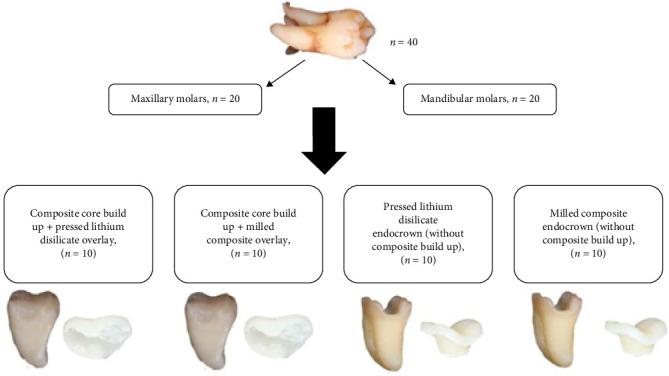
Study groups.

All teeth were subdivided into four subgroups (*n* = 10 per group including five maxillary and five mandibular molars): composite core build up and pressed OL; composite core build up and milled OC; pressed EL without core build up; milled EC without core build up (Figure 2).

**Figure 3:**
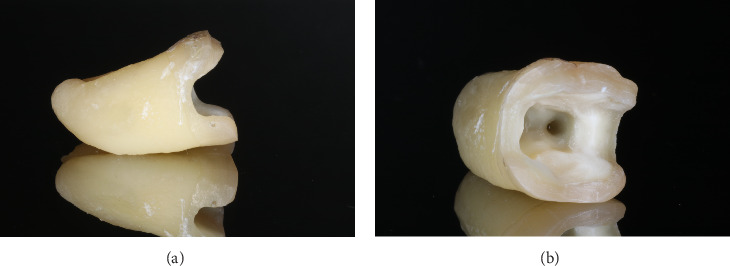
(a) Preparation of specimen from proximal point of view. (b) Preparation of specimen from occlusal point of view.

For indirect restorations teeth were prepared using diamond burs (round 801 green 016; 881 green and red 014; DabDental) and water cooling following guidelines [5]. An occlusal reduction of 1.5–2.0 mm was performed. MOD cavities were created, maintaining the thickness of functional cusps at 2.5–3.0 mm and supporting cusps at 2.0–3.0 mm. Functional cusps were prepared with a 1 mm rounded shoulder margin, leaving at least 1.5–2.0 mm thick cusp tips, while supporting cusps were prepared with a butt joint margin. The proximal cavity floor was prepared 1.0 mm above the CEJ with a butt joint margin, and the mesiodistal and buccolingual cavity widths were 1.5–2.0 mm and 3.0–4.0 mm, respectively. All line angles were smoothed, undercuts were avoided, and the divergence angle of the MOD cavity walls was approximately 6° (Figure 3a, b).

**Figure 4:**
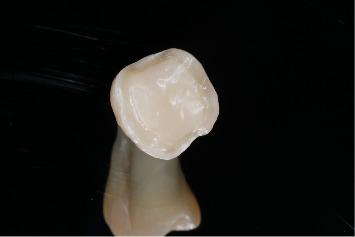
Specimen with core build up.

For the OL and OC groups, the cavities were etched with 35% phosphoric acid (I-GEL Acid Etch Gel 37%; i-Dental) for 15 s, followed by a 15 s water rinse. A bonding agent (Optibond FL; Kerr) was then applied and light cured. The composite core build-up (G-aenial PA-2; GC) was created, light cured, and polished. Proximal cavities (boxes) were reprepared according to the same parameters as previously described (Figure 4).

### 2.4. Restoration Fabrication Process

**Figure 5:**
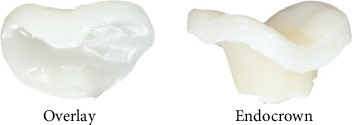
Restorations of overlay and endocrown.

All selected teeth were scanned with a 3Shape Trios 3 dental scanner, and the STL files were sent to a dental laboratory (Institute of Stomatology, Riga, Latvia) where overlay and endocrown restorations were digitally designed to cover all cusps with a uniform thickness of approximately 1.5 mm (Figure 5). Using CAD/CAM technology, overlays and endocrowns were fabricated from pressed lithium disilicate (IPS e.max press; Ivoclar Vivadent) and milled composite resin (Bredent brecam.HIPC; Bredent GmbH and Co. KG).

### 2.5. Cementation

The restorations were inspected and luted using Panavia V5 (Kuraray, Noritake) resin cement. Composite resin restorations were cleaned (Katana cleaner; Kuraray, Noritake), rinsed, and air-dried, while lithium disilicate restorations were etched with 9.5% hydrofluoric acid (Yellow porcelain etch; Cerkamed) for 20 s, rinsed, and air-dried. Both types of restorations were treated with a primer (Clearfil Ceramic Primer plus; Kuraray, Noritake), and the teeth were etched with 35% phosphoric acid (I-GEL Acid Etch Gel 37%; i-Dental) for 30 s, rinsed, air-dried, and primed (Tooth primer; Kuraray, Noritake). Using a mixing tip, resin cement was applied, and the restorations were manually placed. The excess cement after repeated finger pressure was removed with a brush, followed by light curing of the margins for 3–5 s. Residual cement was then removed with a surgical blade (Number 15; Swann-Morton) and the restorations underwent the final light-curing for 20 s per surface and left to set for 4 min. Finally polishing with red and yellow diamond burs and rubber ceramic polishers (StarGloss; Edenta, in the sequence of blue, pink, gray) was performed before restorations being placed in distilled water.

### 2.6. Thermocycling and Preloading

The teeth were stored in a wet unit and sent to Malmö University for thermocycling and fracture strength testing. Specimens underwent 10,000 thermocycles between 5 and 55°C, lasting 60 s per cycle (20 s in each bath, transfer time 10 s) (Thermocycler; SD Mechatronik GmbH). This process was repeated before and after mechanical cyclic loading (MCL). After the initial thermocycling, specimens were set at a 10° inclination in sample holders using epoxy resin (EpoFix; Struers A/S) and mounted in a chewing simulator (CS-4.8; SD Mechatronik GmbH) for 600,000 cycles at 1.63 Hz, using a 5 mm stainless-steel ball under a 50 N load in distilled water 20 ± 3°C. The load was applied in a sliding motion, beginning at the mesiobuccal cusp's inclination and moving 0.5 mm vertically and 0.4 mm horizontally towards the central fissure, followed by unloading and returning to the starting position, which was verified with occlusal foil. After MCL, the specimens underwent a second thermocycling round. If debonding occurred (*n* = 11), restorations were recemented, and thermocycling resumed from the point of debonding.

### 2.7. Fracture Strength Test

After thermocycling and preloading, the specimens underwent a fracture strength test. Each was mounted at a 10° inclination in a Universal Testing System (Instron 4465 with Instron Bluehill Software version 2.35.917; Norwood). A 5 mm steel ball was placed on the occlusal mark at the mesiobuccal cusp's inclination. The loading rate was 0.5 mm/min, and the test ended upon detecting a crack or fracture, with the force recorded in Newtons (N). The test was conducted blindly by a single operator and afterwards the teeth were visually evaluated blindly by three independent operators.

### 2.8. Visual–Microscopic Inspection

After fracture strenght test, the teeth were returned to Riga Stradiņš University and analyzed blindly by two independent operators. The investigation began with a visual examination for cracks or fractures, followed by the removal of the restorations and staining with methylene blue and microscopic examination (OPMI pico; Carl Zeiss) at a magnification of 12.5 × 2.5 to identify additional cracks. The depth of cracks was determined by drilling with a round diamond bur. Cracks and fractures were assessed for their location in enamel, dentin, or cement and classified as either repairable or catastrophic nonrepairable [29], based on whether they crossed multiple tooth surfaces below the CEJ.

### 2.9. Statistical Analysis

IBM SPSS 27 and Jamovi 2.3.28 were used for descriptive statistics. Cross-tabulation summarized and compared crack and fracture occurrences among the four restoration groups (OC, OL, EC, and EL). Nonparametric tests were used due to data distribution. The Kruskal–Wallis test assessed differences in fracture resistance, fracture strength, and catastrophic tooth fractures among groups. While the Mann–Whitney *U* test compared fracture patterns. Fisher's exact test evaluated associations between categorical variables, such as restoration type or restoration material and fracture occurrence. Binomial logistic regression assessed the likelihood of catastrophic fractures based on restoration type and materials, calculating odds ratios (ORs) with 95% confidence intervals (CIs). Statistical significance was set at *p* < 0.05. Although nonparametric tests (Kruskal–Wallis and Mann–Whitney *U*) were used due to non-normal data distribution, an approximate power analysis was conducted using a one-way ANOVA model to estimate statistical power. With a sample size of *n* = 10 per group (total *n* = 40) and a significance level of *α* = 0.05, the study had approximately 53% power to detect a medium effect size (Cohen's *f* = 0.25) and 87% power for a large effect size (Cohen's *f* = 0.40).

## 3. Results

### 3.1. Visual Inspection of the Specimens

**Figure 6:**
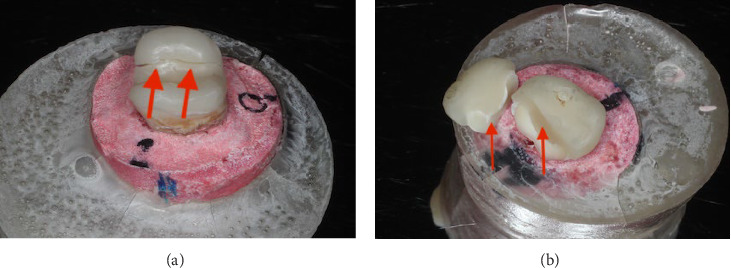
(a) Crack in restoration from mesial to distal side. (b) Catastrophic tooth fracture with restoration fracture.

Visual examination revealed cracks and fractures in all restoration groups after fracture strength test, with the highest frequency (*n* = 5) in the EC group (Figure 6a and Table 1) and the highest frequency (*n* = 9) of restoration fractures in the OL group. (Figure 6b and Table 1). Cracks in the underlying tooth were also noted in all groups with the highest frequency (*n* = 6) in the EL group (Table 2). The pattern of cracks extending to cement was more prevalent in the EL group (Table 2). Furthermore, fractures in the underlying tooth were observed in two groups: EC (*n* = 1); EL (*n* = 4), with the highest frequency in the EL group (Table 2).

### 3.2. Microscopic Inspection of the Specimens

**Figure 7:**
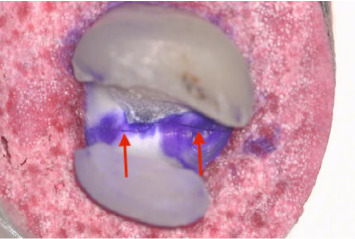
Catastrophic tooth crack from mesial to distal side (×25).

**Figure 8:**
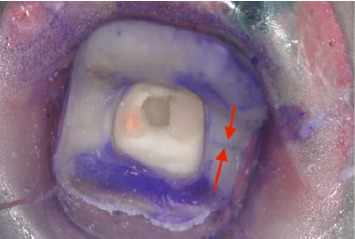
Crack in enamel and dentin (×25).

Microscopic examination showed tooth cracks in all groups, with the OL group having the highest frequency (*n* = 8) (Figures 7 and 8, Table 3). Fractures were observed in the EC and EL groups, with EC having the most (*n* = 3) (Table 3). Catastrophic nonrepairable tooth cracks and fractures were found in the OC, EL, and EC groups, with EC having the highest frequency (*n* = 7) (Table 4).

### 3.3. Results of Fracture Strength Test

**Figure 9:**
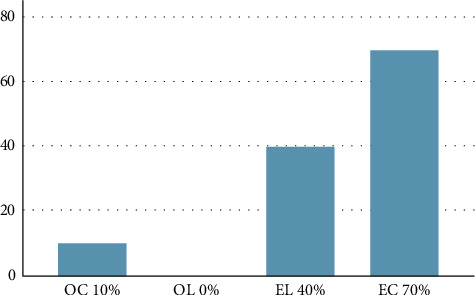
Catastrophic tooth fracture per group (%). EC, composite endocrown; EL, lithium disilicate endocrown; OC, composite overlay; OL, lithium disilicate overlay.

The average fracture resistance strength for each group was: OC-1735.80 N; OL-1709.80 N; EC-1630.00 N; EL-1718.60 N (Table 5). There were no statistically significant differences in fracture resistance strength among the four groups (Kruskal–Wallis test, *p*=0.417) (Table 5). However, the highest individual fracture strength was recorded in the EL group (3770 N). No statistically significant differences were found in fracture patterns across restoration types relative to the fracture strength test (Mann–Whitney *U* test, *p*=0.361) or between groups in terms of fracture strength (N) and catastrophic tooth fractures (Kruskal–Wallis test, *p*=0.247). No statistically significant differences were found across study groups (Fisher's exact test, *p*=0.212), restoration types (Fisher's exact test, *p*=0.191), or restoration materials (Fisher's exact test, *p*=0.337). A statistically significant difference was observed between groups regarding catastrophic tooth fractures (Fisher's exact test, *p*=0.002). The highest frequency of catastrophic tooth fractures was in the EC group (70%), followed by the EL (40%) and OC group (10%), with no occurrences in the OL group (0%) (Figure 9). The binomial logistic regression analysis indicated that endocrowns had a statistically significantly 22 times higher likelihood of catastrophic tooth fractures compared to overlays (OR 22.928, *p*=0.005). Specifically, the EC group was 20 times more likely to experience catastrophic fractures compared to the OC group (OR 20.320, *p*=0.017). (Tables 6 and 7). The binomial logistic regression analysis showed that there was no statistically significant difference between the different restoration materials (lithium disilicate versus composite resin) in terms of the likelihood of catastrophic tooth fracture (*p*=0.102) (Table 8).

## 4. Discussion

The study accepted both null hypotheses, finding no significant difference in fracture resistance among the four groups of endodontically treated teeth. Although not statistically significant, overlay groups showed higher average tooth fracture resistance compared to endocrown groups. This may be due to the reinforcing effect of the composite core build-up, which improves stress distribution because of its lower elastic modulus. This aligns with the previous studies suggesting that composite core build-ups enhance stress distribution and fracture resistance [21]. The EL group displayed a wide range of fracture strength values, exceeding typical clinical scenarios [30–33]. Clinically, such high values are unlikely, even in individuals with increased bite forces due to factors like bruxism [34, 35]. Furthermore, one specimen in the EL group withstood an exceptionally high load (3770 N), which exceeds clinically relevant thresholds and may be attributed to in vitro overloading artifacts. Moreover, the mean fracture strength values showed high variability due to a few individual specimens with markedly higher values, as observed in the EL group. In contrast, the OL group exhibited smaller deviations, as the differences between the minimum and maximum values were not as pronounced. These differences could be explained by the fact that, in the OL group, a composite build-up and a uniform overlay were present, which allowed for a more even distribution of forces.

Despite similar fracture resistances, significant differences were observed in catastrophic tooth fracture rates, with the highest frequency in teeth restored with endocrowns, especially in the EC group (70%). Teeth restored with endocrowns exhibited a statistically significantly 22 times higher likelihood of catastrophic tooth fractures compared to those restored with overlays. Specifically, the EC group was 20 times more likely to experience catastrophic fractures than the OC group. These findings highlight the critical role of restoration design in fracture outcomes, consistent with conclusions from Vervack et al. [36]. The higher catastrophic fracture rate in endocrown groups may be due to stress concentration in the pulpal cavity [16]. Although lithium disilicate has a higher elastic modulus and endocrowns made of lithium disilicate were reported to have more catastrophic fractures, our study found that catastrophic fractures were more common in composite resin endocrowns [37]. The lower elastic modulus of composite resin provides greater flexibility and allows it to withstand higher fracture strength, which may contribute to the increased incidence of catastrophic failure. Studies suggest that deeper pulp chamber extensions (2–4 mm) may increase fracture risk [38, 39], with 3 mm depth generating higher stress under horizontal loading. Endocrowns without a ferrule also exhibit more catastrophic failures [40, 41], emphasizing the importance of careful treatment planning and preparation design for successful restorations on endodontically treated molars. Another factor contributing to the higher fracture frequency and greater incidence of catastrophic failures in endocrowns is the preservation of at least one coronal wall [42, 43].

Our findings align with literature indicating that teeth restored with endocrowns often exhibit a mesiodistal wedge opening fracture pattern due to stress concentration within the pulpal cavity [44–46]. Some studies suggest that choice of the lining material for the pulpal floor affects mechanical outcomes; flexible materials like composite resin lower stress in the cement layer but increase it in the restoration, making material stiffness crucial in stress distribution [47].

The performance differences between composite resin and lithium disilicate restorations come from their distinct material properties, such as elastic modulus and fracture toughness. Glass ceramics like lithium disilicate have a high elastic modulus (~65 GPa), providing better structural integrity but potentially transferring more stress to the tooth. In contrast, composite resin with lower elastic moduli (8–15 GPa) [48, 49], better absorb and distribute stress, which may explain their higher tooth fracture resistance in overlay groups. In terms of fracture toughness, lithium disilicate shows a value of around 3 MPa√m, while composite resin range from 1 to 2 MPa√m [50, 51].

Under axial loading, both lithium disilicate and composite resin endocrowns perform similarly, but under lateral loading, composite resin endocrowns exhibit lower failure rates [52]. Our study found that EC groups demonstrated higher tooth fracture resistance than EL, highlighting the material's stress-absorbing nature. However, catastrophic fractures were more common in EC than in EL, which may be due to the higher deformation capacity of composite materials under unrealistic high loads used in vitro [53, 54]. This suggests that while ECs can endure greater loading, they may also lead to more catastrophic fractures.

Endocrowns, especially those without a ferrule, tend to have more irreparable fractures compared to crowns or other materials like monolithic zirconia [55–58]. While endocrowns are less time-consuming and technique-sensitive to prepare, our research suggests that overlays, particularly those made from lithium disilicate, are superior for preserving tooth structure. The OL group showed no catastrophic fractures, making OLs the optimal choice for restoring endodontically treated molars [59–62]. The absence of catastrophic fractures in the OL group can be explained by the distributed occlusal forces more evenly across the tooth and restoration. However, in our study OC group exhibited only a single catastrophic fracture, suggesting that the design of the restoration may have greater inlfuence on fracture behavior than the material itself.

Identifying and interpreting cracks in restorations is crucial. Our study found discrepancies between visual and microscopic examinations, with microscopic analysis revealing more cracks, especially in the overlay groups (Figures 7 and 8). This emphasizes the importance of thorough microscopic assessments to detect subtle cracks that could compromise tooth longevity [63].

Thermocycling was utilized to simulate the aging of dental materials, subjecting samples to 20,000 cycles to replicate approximately 2 years of clinical conditions [64]. While there is no standardized protocol for laboratory testing, our chosen thermocycling protocol aimed to balance replicating long-term clinical conditions with experimental feasibility [65–67].

Fractures extending apically beyond the CEJ were considered nonrepairable and classified as catastrophic tooth fractures. From a clinical perspective, OCs or ECs are easier to repair in the event of a crack or restoration fracture. In such cases, the affected area can be cleaned, drilled down to the tooth structure, and refilled. In contrast, repairing lithium disilicate restorations is more complex. If the restoration has a superficial crack, polishing is usually sufficient. However, if the crack is deeper or there is a restoration fracture but the tooth is asymptomatic, the area is polished and monitored. In some cases, repair can be done following the bonding protocol with composite resin [68]. If symptoms are present, the entire restoration must be replaced, and it is necessary to assess whether the fracture has extended into the tooth itself.

During thermocycling, 11 specimens experienced debonding, all made from the same composite material, suggesting that improper preparation or material properties may have contributed. All specimens that experienced debonding were recemented following the original cementation protocol, and thermocycling was resumed from the point of interruption. Nevertheless, the specimens continued in the study as originally planned. The debonding was attributed to the possibility that composite may bond less effectively to dentin due to a small amount of resin components in certain material (Bredent brecam.HIPC; Bredent GmbH and Co. KG) [69]. Therefore, it is recommended to bond it with an MDP-containing self-adhesive resin (e.g., dual-hardening adhesive) to achieve better adhesion [70]. Moreover, considering that it was mostly the composite resin overlays that debonded, this explains the loss of retention and the inadequate adhesion to dentin.

Another limitation of this study is the small sample size due to strict inclusion criteria and limited biological material. The lack of blinding in visual–microscopic analysis and the potential for operator bias are also a limitation. Future studies should include larger sample sizes, stratify sample by arch, diverse materials, and preexperiment microscopic evaluations to enhance the reliability and comprehensiveness of the findings.

## 5. Conclusions

Within the limitations of this in vitro study, the following conclusions can be drawn:

1. The type of restoration (overlay or endocrown) does not significantly impact the overall tooth fracture resistance strength.
2. However, overlays may be capable of withstanding higher chewing forces compared to endocrowns.
3. Choosing endocrowns, particularly those made of composite resin, instead of overlays in clinical practice, highlights the potential risk associated with catastrophic tooth fractures.
4. Overlays, particularly those made of lithium disilicate, may demonstrate promising results in clinical practice with no risk of catastrophic tooth fractures.
5. It seems that the choice between restorative material (lithium disilicate or composite resin) does not appear to influence the risk of catastrophic tooth fractures.

## Figures and Tables

**Table 1:** Visual inspection of restoration cracks and fracture after fracture strength test.

Group	Crack in restoration	Fracture in restoration	No cracks or fractures in restoration
OC	*n* = 4	*n* = 6	*n* = 0
OL	*n* = 1	*n* = 9	*n* = 0
EC	*n* = 5	*n* = 4	*n* = 1
EL	*n* = 4	*n* = 6	*n* = 0
Total	*n* = 14	*n* = 25	*n* = 1

Abbreviations: EC, composite endocrown; EL, lithium disilicate endocrown; OC, composite overlay; OL, lithium disilicate overlay.

**Table 2:** Visual inspection of underlying tooth cracks and fracture after fracture strength test.

Group	Crack in enamel	Crack in enamel- dentin	Crack in enamel- cement	Crack in enamel- dentin-cement	Total cracks	Fracture in enamel	Fracture in enamel-dentin	Fracture in enamel-dentin-cement	Total fractures
OC	*n* = 0	*n* = 1	*n* = 1	*n* = 0	*n* = 2	*n* = 0	*n* = 0	*n* = 0	*n* = 0
OL	*n* = 3	*n* = 1	*n* = 0	*n* = 1	*n* = 5	*n* = 0	*n* = 0	*n* = 0	*n* = 0
EC	*n* = 2	*n* = 0	*n* = 1	*n* = 2	*n* = 5	*n* = 0	*n* = 0	*n* = 1	*n* = 1
EL	*n* = 1	*n* = 0	*n* = 5	*n* = 0	*n* = 6	*n* = 0	*n* = 0	*n* = 4	*n* = 4
Total					*n* = 18				*n* = 5

Abbreviations: EC, composite endocrown; EL, lithium disilicate endocrown; OC, composite overlay; OL, lithium disilicate overlay.

**Table 3:** Microscopic inspection of repairable tooth cracks and fractures after fracture strength test.

Group	Crack in enamel	Crack in dentin	Crack in enamel -dentin	Crack in enamel- dentin-cement	Total cracks	Fracture in enamel	Fracture in enamel-dentin	Fracture in enamel-dentin-cement	Total fractures
OC	*n* = 2	*n* = 0	*n* = 3	*n* = 0	*n* = 5	*n* = 0	*n* = 0	*n* = 0	*n* = 0
OL	*n* = 3	*n* = 1	*n* = 2	*n* = 2	*n* = 8	*n* = 0	*n* = 0	*n* = 0	*n* = 0
EC	*n* = 0	*n* = 0	*n* = 2	*n* = 0	*n* = 2	*n* = 0	*n* = 0	*n* = 3	*n* = 3
EL	*n* = 0	*n* = 0	*n* = 1	*n* = 1	*n* = 2	*n* = 0	*n* = 0	*n* = 1	*n* = 1
Total					*n* = 17				*n* = 4

Abbreviations: EC, composite endocrown; EL, lithium disilicate endocrown; OC, composite overlay; OL, lithium disilicate overlay.

**Table 4:** Microscopic inspection of catastrophic nonrepairable tooth cracks and fractures after fracture strength test.

Group	Catastrophic crack	Catastrophic fracture	Total
OC	*n* = 1	*n* = 0	*n* = 1
OL	*n* = 0	*n* = 0	*n* = 0
EC	*n* = 4	*n* = 3	*n* = 7
EL	*n* = 3	*n* = 1	*n* = 4
Total			*n* = 12

Abbreviations: EC, composite endocrown; EL, lithium disilicate endocrown; OC, composite overlay; OL, lithium disilicate overlay.

**Table 5:** Descriptives of fracture strength test per group.

Group	Mean (N) ± SD	Minimum (N)	Maximum (N)
OC	1735.80 ± 117.192	1181	2452
OL	1709.80 ± 92.374	1262	2062
EC	1630.00 ± 141.352	1107	2277
EL	1718.60 ± 267.882	958	3777

Abbreviations: EC, composite endocrown; EL, lithium disilicate endocrown; OC, composite overlay; OL, lithium disilicate overlay.

**Table 6:** Possibility of catastrophic tooth fracture between groups.

Predictor	*p*-Value	Odds ratio	95% Confidence interval for mean	
			Lower bound	Upper bound
OL/OC	0.994	7.74	0.00	Inf
EC/OC	0.017	20.32	1.70	241.81
EL/OC	0.155	5.86	0.511	67.38
Fracture strength test (N)	0.482	0.99	0.99	1.00

Abbreviations: EC, composite endocrown; EL, lithium disilicate endocrown; OC, composite overlay; OL, lithium disilicate overlay.

**Table 7:** Possibility of catastrophic tooth fracture between different types of restorations (endocrowns/overlays).

Predictor	*p*-Value	Odds ratio	95% Confidence interval for mean	
			Lower bound	Upper bound
EL and EC/OC and OL	0.005	22.928	2.53567	207.31
Fracture strength test (N)	0.447	0.999	0.99803	1.00

Abbreviations: EC, composite endocrown; EL, lithium disilicate endocrown; OC, composite overlay; OL, lithium disilicate overlay.

**Table 8:** Possibility of catastrophic tooth fracture between different restoration materials (lithium disilicate/composite).

Predictor	*p*-Value	Odds ratio	95% Confidence interval for mean	
			Lower bound	Upper bound
OL and EL/OC and EC	0.102	0.293	0.0673	1.28
Fracture strength test (N)	0.290	0.999	0.9972	1.00

Abbreviations: EC, composite endocrown; EL, lithium disilicate endocrown; OC, composite overlay; OL, lithium disilicate overlay.

## Data Availability

The datasets generated during the current study are available from the corresponding author upon reasonable request.
